# Twisted microdomains in liquid crystals for polarization-insensitive phase modulation

**DOI:** 10.1038/s41377-023-01351-8

**Published:** 2024-01-05

**Authors:** Yifei Ma, Zimo Zhao, Stephen M. Morris, Chao He

**Affiliations:** Department of Engineering Science, University of Oxford, Oxford, OX1 3PJ UK

**Keywords:** Optics and photonics, Optical physics

## Abstract

Polarization-independent phase modulators based upon liquid crystals (LCs) with a simple device architecture have long been desired for a range of optical applications. Recently, researchers have demonstrated a novel fabrication procedure using cholesteric LCs as a primer for achieving low polarization dependence coupled with a large phase modulation depth.

Spatial light modulators (SLMs) based on liquid crystals (LCs) have been widely used in a range of modern optical applications for sophisticated optical phase profile manipulation, such as structured beam generation, and adaptive optics^1–5^. However, unlike other polarization-insensitive phase manipulating devices such as deformable mirrors (DMs) or digital micromirror devices (DMDs), the behaviour of these LC phase modulators depends upon the state of polarization (SoP) of the light beam when deployed in real-world applications^5,6^. Thus, polarizers are often added to the SLM to achieve a designed modulation effect, thereby limiting the overall optical efficiency. Developing solutions that enable the realization of polarization-insensitive LC phase modulators is therefore highly desirable as these materials offer benefits that are not readily realized with alternative technologies such as DMs and DMDs^7^. Broadly, there are two separate potential routes to developing polarization-insensitive LC phase modulators: one approach is to employ the use of LC phases that self-organize to form a polarization-insensitive structure, such as blue phase LCs^8,9^. However, in such cases, the high driving voltage is typically rather prohibitive and often incompatible with existing CMOS backplane technologies^9^. An alternative approach is to carefully control the spatial alignment of the LC director; unfortunately, achieving high-resolution spatially localized control of the LC alignment is challenging, typically requiring complex structure designs and alignment procedures^10^.

In a recent article^11^, Mingyuan Tang and co-authors have proposed a new polarization-insensitive LC phase modulator that contains multiple microdomains in a single-layered device. An innovative light-controlled azimuth angle process utilizing the optical rotatory effect in cholesteric LCs (CLC) has been developed to create multi-microdomain orthogonal twisted structures. Each microdomain contains a twisted LC structure with adjacent orthogonal-oriented domains. To create the twisted microdomains, the authors have creatively used the CLC as the primer for orientating the photoalignment layers. To ensure that the orientation of the easy axis of the photoalignment layers between the two substrates is rotated by the desired angle, the CLC mixture was carefully designed to obtain the required pitch of the helix for the incident wavelength employed in the study. Such an approach provides a novel way of manufacturing micro-domains containing different orientations of the alignment layers using a simple checkerboard photomask to make the phase modulator polarization insensitive. To realize the patterned twisted microdomain structure (referred to as the multi-microdomain orthogonal twisted (MMOT) structure), the CLC is replaced with a nematic LC layer (Fig. 1). Results indicate that the MMOT structure shows a polarization dependence of less than 10% with 3.4π phase retardation modulation, which is particularly promising for potential applications necessitating polarization insensitivity.

**Fig. 1:**
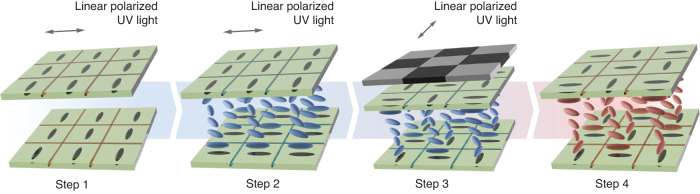
A diagram demonstrating the principle of the fabrication process, where CLC is used as the primer for orientating photoalignment layers in an orthogonal manner (Steps 1–3) and nematic LC replaces CLC in multi-microdomain orthogonal twisted (MMOT) structures (Step 4)

With the assistance of the newly proposed fabrication method, it is possible to construct a more complex in-plane alignment structure with various profiles, which is traditionally difficult to achieve with a single mask alongside relatively few exposures to blanket illumination of UV light. The approach provides an important fabrication strategy for future LC configurations. The high phase retardation, simple device architecture, as well as flexibility of the grid size of the device, are of benefit in modern adaptive optics application areas, such as in microscopy for deep tissue imaging, where aberrations can be large thus requiring large modulation capability^5,12^. Furthermore, this fabrication approach also provides scope for a compact, insertable phase control device, which may benefit applications such as optical communications. Further work may involve providing pixelated control of the LC as well as the demonstration of full control of the SoP of the light field. For future applications, the response time of the phase modulator will also be of importance, although this is not discussed explicitly in the report, it again fits well in the pipeline of future investigations. In summary, the present work highlights a novel way for a polarization-independent phase modulator using LCs paving the way for a variety of applications in optics and photonics.
